# Beyond Glucose Control: A Comprehensive Review of Sodium-Glucose Cotransporter 2 (SGLT2) Inhibitors in Heart Failure, Chronic Kidney Disease, and Nonalcoholic Fatty Liver Disease

**DOI:** 10.7759/cureus.104564

**Published:** 2026-03-02

**Authors:** Alexander R Sosa, Michael C Sosa

**Affiliations:** 1 Burnett School of Biomedical Sciences, University of Central Florida, Orlando, USA; 2 College of Arts and Sciences, University of South Florida, Tampa, USA

**Keywords:** cardiorenal protection, cardiovascular outcomes, chronic kidney disease, diabetic nephropathy, heart failure, hfpef, nafld, precision medicine, sglt2 inhibitors, type 2 diabetes

## Abstract

Sodium-glucose cotransporter 2 (SGLT2) inhibitors were first introduced to enhance glycemic control in type 2 diabetes mellitus (T2DM). However, a series of large-scale cardiovascular and renal outcomes trials have revealed their substantial benefits in reducing heart failure (HF) hospitalizations, slowing chronic kidney disease (CKD) progression, and improving cardiorenal outcomes, even among non-diabetic populations. Early studies further suggest potential benefits in non-alcoholic fatty liver disease (NAFLD). In this comprehensive review, we synthesize the mechanistic basis, landmark trials, real-world evidence, and emerging guidelines that position SGLT2 inhibitors as cornerstone therapies in the management of HF and CKD, with growing research in NAFLD. By highlighting key clinical outcomes, safety profiles, and cost-effectiveness considerations, we underscore how SGLT2 inhibitors transcend their original role in glucose regulation to provide multifaceted protection across the cardiorenal-metabolic spectrum.

## Introduction and background

Sodium-glucose cotransporter 2 (SGLT2) inhibitors reduce renal glucose reabsorption by inhibiting SGLT2 in the proximal tubule, thereby increasing urinary glucose excretion and modestly lowering hemoglobin A1c in type 2 diabetes mellitus (T2DM); early outcomes data established cardiovascular safety, and contemporary guidelines now recommend SGLT2 inhibitors for organ protection in chronic kidney disease (CKD) at appropriate estimated glomerular filtration rate (eGFR) thresholds [1-3]. In addition to lowering glucose, the same proximal tubular effect increases distal sodium delivery, producing natriuresis and osmotic diuresis that can reduce plasma volume, blood pressure, and ventricular filling pressures, mechanisms directly relevant to heart failure (HF) and CKD [3]. Large cardiovascular and renal outcomes trials initially conducted for safety and efficacy in diabetes, including EMPA-REG OUTCOME, DAPA-HF, and CREDENCE, as well as subsequent HF- and kidney-focused trials, demonstrated clinically meaningful reductions in HF hospitalization and kidney disease progression that extend to many non-diabetic populations [2,4-7]. Early cardiovascular outcomes trials (CVOTs) across the class, including EMPA-REG OUTCOME, CANVAS, and VERTIS CV, demonstrated cardiovascular safety and suggested heterogeneity in atherosclerotic outcomes, whereas subsequent dedicated HF and CKD trials demonstrated more consistent reductions in HF hospitalization and kidney disease progression, including in many non-diabetic populations [2,4-10]. Although renal hemodynamic and natriuretic effects were hypothesized to be beneficial, the magnitude and consistency of cardiorenal outcome benefits across trials were initially greater than expected.

HF is a syndrome of impaired cardiac pumping and/or filling that leads to symptoms and repeated hospitalizations, while CKD is defined by persistent reductions in eGFR, albuminuria, or other markers of kidney damage with elevated cardiovascular risk. SGLT2 inhibitors are now considered unique among cardiometabolic therapies because they provide organ-protective benefits across the cardiorenal axis that are not fully explained by glycemic effects alone [4-7].

Emerging evidence also suggests that SGLT2 inhibitors may improve hepatic steatosis and inflammatory activity in metabolic dysfunction-associated steatotic liver disease (MASLD, formerly non-alcoholic fatty liver disease (NAFLD)) and metabolic dysfunction-associated steatohepatitis (MASH, formerly nonalcoholic steatohepatitis (NASH)), although the overall evidence base remains smaller and more heterogeneous than for HF and CKD [11,12]. Current NAFLD data are limited to small, randomized trials and surrogate markers rather than hard hepatic outcomes. In this narrative review, we synthesize mechanistic rationale, landmark trials, real-world evidence, and guideline positioning for SGLT2 inhibitors in HF and CKD, and we summarize the current state of evidence in hepatic disease, including key limitations and research gaps. We also address implementation barriers, including out-of-pocket costs and formulary restrictions.

## Review

Methods and literature search

We conducted a structured literature search to identify clinical trials, observational studies, meta-analyses, and guideline documents evaluating SGLT2 inhibitors in HF, CKD, and hepatic steatosis/steatohepatitis. Databases searched included PubMed/MEDLINE and Google Scholar. For Google Scholar, we screened the first 200 results per query, sorted by relevance to capture highly cited clinical and guideline literature. Searches were supplemented by hand-searching reference lists of high-impact trials and guideline documents. The primary search window spanned January 2014 through December 2025 to capture contemporary outcome trials and guideline updates; seminal earlier mechanistic or disease-defining references were included when necessary for context.

Search concepts were combined using Boolean operators, typically using OR within concept groups and AND across concept groups (examples provided in Table 1). Eligibility criteria emphasized: (1) randomized controlled trials (RCTs) and dedicated outcomes trials for HF and CKD, (2) large real-world studies and registries where RCT data were limited, (3) hepatic studies that reported imaging-based liver fat outcomes, biochemical markers, or histological endpoints, and (4) major society guidelines and consensus statements. Non-English articles, preclinical-only studies, and studies without sufficient outcome reporting were excluded unless necessary to explain key mechanisms.

**Table 1:** Example literature search concepts and keywords used for this review. SGLT2: sodium-glucose cotransporter 2, CKD: chronic kidney disease, HFrEF: heart failure with reduced ejection fraction, HFpEF: heart failure with preserved ejection fraction, HFmrEF: heart failure with mildly reduced ejection fraction, NAFLD: non-alcoholic fatty liver disease, MASLD: metabolic dysfunction-associated steatotic liver disease, ESKD: end-stage kidney disease, DKA: diabetic ketoacidosis, UACR: urine albumin-to-creatinine ratio, MRI-PDFF: MRI-derived proton density fat fraction, eGFR: estimated glomerular filtration rate

Concept	Example literature search concepts and keywords used
SGLT2 inhibitors (general)	(SGLT2 inhibitor* OR SGLT2i OR “sodium-glucose cotransporter 2” OR dapagliflozin OR empagliflozin OR canagliflozin OR ertugliflozin)
Heart failure outcomes	(heart failure OR HFrEF OR HFpEF OR HFmrEF OR “reduced ejection fraction” OR “preserved ejection fraction” OR hospitalization OR “cardiovascular death” OR DAPA-HF OR EMPEROR OR DELIVER)
Kidney outcomes	(chronic kidney disease OR CKD OR diabetic kidney disease OR eGFR OR albuminuria OR UACR OR “albumin-to-creatinine ratio” OR ESKD OR CREDENCE OR DAPA-CKD OR EMPA-KIDNEY)
Hepatic steatosis/steatohepatitis	(NAFLD OR MASLD OR “fatty liver” OR “hepatic steatosis” OR NASH OR MASH OR MRI-PDFF OR “proton density fat fraction” OR “liver fat fraction” OR fibrosis OR biopsy OR steatohepatitis)
Safety	(euglycemic diabetic ketoacidosis OR “euglycemic DKA” OR genital infection OR mycotic infection OR volume depletion OR hypotension OR acute kidney injury OR amputation OR fracture OR “Fournier gangrene”)
Guidelines and implementation	(guideline OR consensus OR “practice guideline” OR ACC OR AHA OR HFSA OR ESC OR KDIGO OR “American Diabetes Association”)
Implementation and economics	(cost OR cost-effectiveness OR affordability OR access OR adherence OR persistence OR formulary OR “prior authorization” OR “out-of-pocket”)

Study selection and synthesis were qualitative. We prioritized higher levels of evidence (outcomes trials and meta-analyses) for clinical conclusions and used mechanistic and smaller studies to contextualize biological plausibility. Because this is a narrative review rather than a formal systematic review or meta-analysis, we did not perform quantitative pooling of effect estimates. To increase transparency, we report core trial characteristics in Table 2 and explicitly note when evidence is preliminary or surrogate-based, particularly for hepatic outcomes. No formal risk-of-bias tool was applied because this is a narrative review; we prioritized outcomes trials and society guidelines, and we note limitations of observational data and surrogate endpoints. Searches were last updated in January 2026.

**Table 2:** Key SGLT2 Clinical Trials This table summarizes key clinical trials evaluating the efficacy and safety of SGLT2 inhibitors across different conditions, including heart failure, chronic kidney disease, and non-alcoholic fatty liver disease. SGLT2: sodium-glucose cotransporter 2, T2DM: type 2 diabetes mellitus, CKD: chronic kidney disease, HF: heart failure, HFrEF: heart failure with reduced ejection fraction, HFpEF: heart failure with preserved ejection fraction, CV: cardiovascular, NAFLD: non-alcoholic fatty liver disease, ESKD: end-stage kidney disease, LVEF: left ventricular ejection fraction, DKA: diabetic ketoacidosis, UACR: urine albumin-to-creatinine ratio, MRI-PDFF: MRI-derived proton density fat fraction, eGFR: estimated glomerular filtration rate

Study/Trial Name	Population	Intervention	Outcome(s) Measured	Key Findings	Safety Profile/Adverse Effects	Citation	Category
DAPA-HF (2019)	4,744 patients with HFrEF (LVEF ≤ 40%) ± T2DM	Dapagliflozin (10 mg daily) vs. placebo	HF: HF hospitalization or CV death	26% relative risk reduction (HR 0.74, 95% CI 0.65-0.85)	Genital mycotic infections; volume depletion; rare DKA	[4]	Heart Failure
EMPEROR-Reduced (2020)	3,730 patients with HFrEF (LVEF ≤ 40%) ± T2DM	Empagliflozin (10 mg daily) vs. placebo	HF: HF hospitalization or CV death	25% risk reduction (HR 0.75, 95% CI 0.65-0.86)	Genital mycotic infections; volume depletion; rare DKA	[10]	Heart Failure
EMPEROR-Preserved (2021)	~6,000 patients with HFpEF (LVEF ≥ 40%)	Empagliflozin (10 mg daily) vs. placebo	HF: CV death or HF hospitalization	21% risk reduction (HR 0.79, 95% CI 0.69-0.90)	Genital mycotic infections; volume depletion; rare DKA	[17]	Heart Failure
DELIVER (2022)	>6,000 patients with mildly reduced or preserved EF (≥40%)	Dapagliflozin (10 mg daily) vs. placebo	HF: CV death or worsening HF events	18% relative risk reduction (HR 0.82, 95% CI 0.73-0.92)	Genital mycotic infections; volume depletion; rare DKA	[16]	Heart Failure
DAPA-CKD (2020)	4,304 patients with CKD (eGFR 25-75 mL/min/1.73 m², UACR 200-5000 mg/g), ~32% non-diabetic	Dapagliflozin (10 mg daily) vs. placebo	CKD: CKD progression or CV death	39% risk reduction (HR 0.61, 95% CI 0.51-0.72)	Genital mycotic infections; volume depletion; rare DKA	[6]	Chronic Kidney Disease
EMPA-KIDNEY (2023)	6,609 patients with CKD (eGFR 20-45 or eGFR 45-90 with UACR ≥200 mg/g)	Empagliflozin (10 mg daily) vs. placebo	CKD: CKD progression or CV death	28% risk reduction (HR 0.72, 95% CI 0.64-0.82)	Genital mycotic infections; volume depletion; rare DKA	[7]	Chronic Kidney Disease
CREDENCE (2019)	4,401 patients with T2DM + albuminuric CKD (eGFR 30-90 mL/min/1.73 m²)	Canagliflozin (100 mg daily) vs. placebo	CKD: Composite of ESKD, doubling of serum creatinine, or renal/CV death	30% risk reduction (HR 0.70, 95% CI 0.59-0.82)	Genital mycotic infections; volume depletion; consider foot-care vigilance with canagliflozin given prior amputation signal in CANVAS	[5,8]	Chronic Kidney Disease
E-LIFT Trial	50 patients with T2DM + NAFLD	Empagliflozin (10 mg daily) vs. standard care	NAFLD: Liver fat reduction (imaging surrogate); fibrosis not robustly assessed	Significant reduction in liver fat	Generally well tolerated; genital mycotic infections reported	[11]	NAFLD
Dapagliflozin NAFLD MRI-PDFF trial (Shi et al., 2023)	T2DM + NAFLD patients	Dapagliflozin (10 mg daily)	NAFLD: MRI-PDFF liver fat fraction (imaging surrogate); fibrosis not robustly assessed	Improved MRI-measured liver fat	Generally well tolerated; genital mycotic infections; volume depletion	[12]	NAFLD

We extracted and reported effect estimates (e.g., hazard ratios and 95% confidence intervals) as provided in the original trial publications and guidelines. Because this manuscript is a narrative review, we did not perform meta-analysis, meta-regression, or new subgroup pooling, and we did not compute additional P values beyond those reported in the source studies. Accordingly, our synthesis is descriptive and emphasizes consistency of direction and clinical relevance across outcomes trials, while explicitly distinguishing hard endpoints from surrogate outcomes.

Mechanisms of action and pharmacological rationale

**Figure 1:**
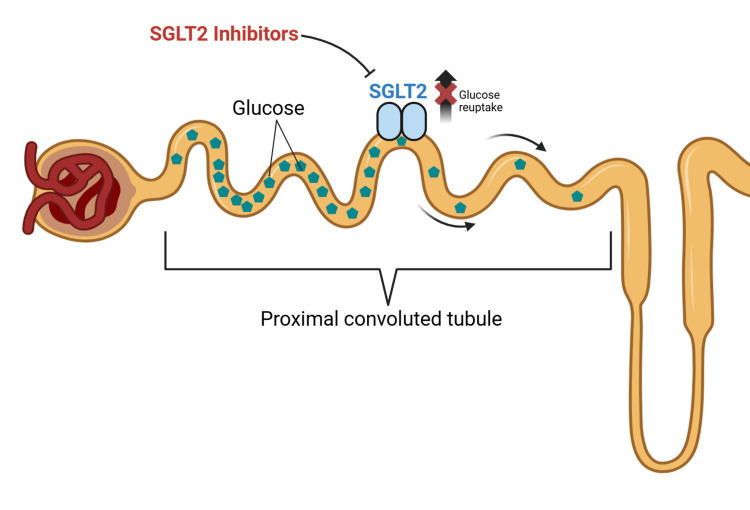
Biochemical Mechanism of Action for Sodium-Glucose Cotransporter 2 (SGLT2) Inhibitors This figure illustrates how SGLT2 inhibitors block glucose reabsorption in the proximal renal tubule, leading to natriuresis, osmotic diuresis, and subsequent cardioprotective and renoprotective effects. Image created by the authors using BioRender.

SGLT2 inhibitors exert cardiometabolic effects through interrelated renal, hemodynamic, and cellular pathways. By inhibiting SGLT2 in the proximal tubule, these agents reduce coupled glucose and sodium reabsorption, increasing urinary glucose and sodium excretion. This results in osmotic diuresis and natriuresis, modest blood pressure reduction, and lower ventricular preload and afterload, providing a plausible physiologic link to HF benefits beyond glycemic control [3]. Additional proposed mechanisms include attenuation of renal hyperfiltration, improved myocardial energy utilization, reduction in adiposity and uric acid, and anti-inflammatory and anti-fibrotic signaling that may contribute to both cardiac and kidney protection (Figure 1) [13,14].

A key renal mechanism is restoration of tubuloglomerular feedback. Increased sodium delivery to the macula densa activates afferent arteriolar vasoconstriction and reduces intraglomerular pressure, thereby mitigating maladaptive hyperfiltration and slowing eGFR decline. Clinically, this mechanism is consistent with the characteristic early, modest dip in eGFR after initiation that stabilizes over time and is followed by a slower long-term rate of kidney function loss in outcomes trials [6]. Human physiologic studies also support this hemodynamic mechanism [15].

Many mechanistic pathways were first described in preclinical models, but multiple effects have also been observed in humans, including reductions in albuminuria, blood pressure, and congestion markers, as well as improved HF outcomes in both diabetic and non-diabetic populations [6,4,16,7]. However, the relative contribution of each mechanism remains uncertain, and several hypotheses (such as ketone-mediated myocardial energetics) may represent secondary adaptations rather than the primary driver of clinical benefit.

SGLT2 inhibitors in heart failure

In heart failure with reduced ejection fraction (HFrEF), large outcomes trials demonstrated that SGLT2 inhibitors reduce the risk of worsening HF events and cardiovascular death when added to standard guideline-directed medical therapy. DAPA-HF showed benefit with dapagliflozin in patients with HFrEF regardless of diabetes status, and EMPEROR-Reduced demonstrated similar efficacy with empagliflozin, supporting a class effect across agents and populations [4,10]. These benefits are biologically plausible given effects on volume unloading, ventricular filling pressures, blood pressure, and neurohormonal stress, alongside potential anti-inflammatory and anti-fibrotic actions [3,14].

For heart failure with preserved ejection fraction (HFpEF) and heart failure with mildly reduced ejection fraction (HFmrEF), EMPEROR-Preserved and DELIVER expanded evidence to patients with left ventricular ejection fraction above the traditional HFrEF threshold. Across these trials, reductions in the composite endpoint were primarily driven by fewer HF hospitalizations, while effects on all-cause or cardiovascular mortality were smaller and may not be statistically significant in some HFpEF subgroups [16,17]. Available subgroup analyses suggest consistent relative benefit across the ejection fraction spectrum, including HFmrEF, supporting guideline incorporation of SGLT2 inhibitors for symptomatic HFpEF and HFmrEF to reduce HF hospitalization and cardiovascular events [18,19].

In contemporary practice, SGLT2 inhibitors are integrated alongside renin-angiotensin-aldosterone system (RAAS) inhibitors, beta blockers, mineralocorticoid receptor antagonists (MRAs), and, when appropriate, angiotensin receptor-neprilysin inhibitors (ARNI). Because these drug classes target complementary pathways, combination therapy may provide additive benefit in HF and CKD, while careful monitoring for volume depletion and renal function changes helps ensure safe implementation [18,19].

Overall, SGLT2 inhibitors are now recognized as a standard component of therapy for both HFrEF and HFpEF, joining other foundational treatments such as RAAS, beta-blockers, and MRAs [20].

SGLT2 inhibitors in chronic kidney disease

Dedicated renal outcomes trials demonstrate that SGLT2 inhibitors slow CKD progression and reduce kidney failure events. CREDENCE established canagliflozin benefit in diabetic kidney disease with albuminuria, while DAPA-CKD and EMPA-KIDNEY broadened evidence to include patients with and without diabetes, showing reductions in sustained eGFR decline, end-stage kidney disease (ESKD), and renal or cardiovascular death endpoints [5-7]. Across trials, benefits were observed on both hard outcomes and intermediate markers, with consistent reductions in albuminuria, a key prognostic marker in CKD [6].

Mechanistically, SGLT2 inhibition reduces proximal sodium reabsorption and restores tubuloglomerular feedback, lowering intraglomerular hypertension and reducing hyperfiltration injury. This is reflected clinically by an expected early decline in eGFR followed by long-term preservation of kidney function and lower rates of ESKD in outcomes trials [6]. Additional contributions may include improved metabolic milieu, reduced inflammation and oxidative stress, and hemodynamic unloading, although the dominant driver of benefit likely varies by patient phenotype [13,14].

Important evidence gaps remain for patients with very advanced CKD (eGFR <20 mL/min/1.73 m²) and for those receiving dialysis, because many pivotal trials excluded these groups or included few such participants. Ongoing studies and post-marketing surveillance will be needed to clarify efficacy and safety in these populations and to refine initiation and continuation thresholds in clinical practice.

Emerging role in metabolic dysfunction-associated steatotic liver disease

MASLD (formerly NAFLD) encompasses hepatic steatosis with metabolic risk factors, while MASH (formerly NASH) represents the inflammatory and fibrosing phenotype with a higher risk of progression. Early trials of SGLT2 inhibitors in hepatic disease have largely focused on surrogate endpoints such as MRI-derived proton density fat fraction (MRI-PDFF), liver enzymes, and non-invasive fibrosis scores. Accordingly, most studies have not robustly assessed fibrosis progression or biopsy-confirmed histologic endpoints (eg, MASH resolution or fibrosis regression), so long-term liver outcome benefits remain uncertain. For example, the E-LIFT trial reported reductions in liver fat with empagliflozin, and a randomized trial by Shi et al. reported reduced liver and pancreatic fat with dapagliflozin in patients with T2DM and hepatic steatosis, alongside improvements in aminotransferases and inflammatory markers [12,11].

While reductions in liver fat and aminotransferases are encouraging, these surrogates do not always translate to improved long-term clinical outcomes. Histological endpoints, including MASH resolution and fibrosis regression, are the most clinically meaningful outcomes but have been relatively sparse in earlier SGLT2 inhibitor studies. More recently, a multicentre, double blind, randomized, placebo-controlled trial in biopsy-diagnosed MASH reported that dapagliflozin increased the proportion of participants achieving MASH improvement without worsening fibrosis, as well as fibrosis improvement without worsening MASH, compared with placebo, strengthening the case that some benefits may extend beyond imaging surrogates [21]. Despite this progress, the role of SGLT2 inhibitors in hepatic disease should still be considered investigational, and longer-term, adequately powered trials are required to define which hepatic phenotypes benefit most and how these agents should be positioned relative to other emerging MASH therapies. Consistent with this, hepatology society guidance (American Association for the Study of Liver Disease (AASLD) and the European Association for the Study of the Liver, European Association for the Study of Diabetes, and European Association for the Study of Obesity (EASL-EASD-EASO) clinical practice guideline) does not recommend SGLT2 inhibitors as liver-directed therapy for MASLD/MASH at this time, and use is generally driven by cardiometabolic and cardiorenal indications [22,23].

Potential hepatic mechanisms include weight reduction and improved insulin sensitivity, which can decrease hepatic de novo lipogenesis and lipotoxicity. Additionally, SGLT2 inhibitors may exert anti-inflammatory and anti-fibrotic effects by reducing proinflammatory cytokines and potentially lowering fibrogenic signaling [13]. They may also modulate hepatic energy balance through changes in substrate utilization (including increased fatty acid oxidation and ketogenesis) and reductions in oxidative stress and systemic inflammatory signaling, which could plausibly influence steatohepatitis and fibrogenesis pathways [13,14]. However, mechanistic evidence in humans remains evolving, and future studies should integrate histology, non-invasive fibrosis markers, and cardiometabolic outcomes to clarify clinical relevance.

Clinical application and guideline implementation

Real-World Effectiveness and Implementation

Multiple observational studies and registries (e.g., CVD-REAL, EMPRISE) report associations between SGLT2 inhibitor use and lower rates of HF hospitalization and cardiovascular events compared with other glucose-lowering therapies, supporting external validity of randomized trial findings [24,25]. These studies broaden generalizability by capturing older patients, comorbidity-heavy populations, and routine-care adherence patterns that are underrepresented in RCTs, and they provide complementary safety signal surveillance in larger, longer-duration cohorts [25,26]. However, real-world studies are susceptible to residual confounding and channeling bias, so they should be interpreted as complementary to outcomes trials rather than definitive evidence of causality. To date, large real-world datasets have not consistently reported MASLD/MASH-specific endpoints (e.g., fibrosis progression or liver outcomes), representing an evidence gap.

Guideline Recommendations and Treatment Positioning

Major guidelines now recommend SGLT2 inhibitors across the HF spectrum. The 2022 American Heart Association/American College of Cardiology/Heart Failure Society of America (AHA/ACC/HFSA) guideline and the 2023 European Society of Cardiology (ESC) focused update endorse SGLT2 inhibitors as foundational therapy to reduce HF hospitalization and improve outcomes across EF phenotypes when appropriate [18,19]. In practice, SGLT2 inhibitors are generally initiated early and used in combination with other guideline-directed therapies (RAAS inhibition or ARNI, beta blockers, and MRAs), with sequencing individualized based on blood pressure, congestion, kidney function, and tolerability [18,19]. In CKD, Kidney Disease: Improving Global Outcomes (KDIGO) (2022) and the American Diabetes Association (ADA) Standards of Care (2025) recommend SGLT2 inhibitors in people with T2DM and CKD above specified eGFR thresholds because they slow CKD progression and reduce cardiovascular events [1,27]. In CKD algorithms, SGLT2 inhibitors function as organ-protective therapy layered onto RAAS blockade (when tolerated), with ongoing monitoring of eGFR and albuminuria to guide risk stratification and follow-up [1,27]. In practice, implementation requires attention to volume status, concomitant diuretics, genital infection counseling, and monitoring of kidney function during initiation, particularly in older adults and those with advanced CKD [1,27]. For hepatic disease, AASLD and EASL-EASD-EASO guidance does not endorse SGLT2 inhibitors as liver-directed therapy; use is primarily driven by cardiometabolic and cardiorenal indications while histology-based liver evidence continues to mature [22,23].

Safety, tolerability, and monitoring

Across trials, the most consistently reported adverse effects of SGLT2 inhibitors are genital mycotic infections and, less commonly, urinary tract infections; these are usually mild and respond to standard therapy with appropriate hygiene counseling [2,8]. Because SGLT2 inhibitors promote osmotic diuresis and natriuresis, patients may experience volume depletion, dizziness, or hypotension, particularly when combined with loop diuretics or in older adults. Amputation and fracture signals were primarily observed with canagliflozin in the CANVAS program and are not clearly a class-wide effect [8]. Clinicians should assess volume status at initiation, consider diuretic dose adjustment when appropriate, and counsel patients on maintaining hydration during intercurrent illness [1,27].

A rare but clinically important risk is euglycemic diabetic ketoacidosis (DKA), most often in patients with diabetes during periods of reduced insulin intake, prolonged fasting, acute illness, or perioperative stress [28]. Risk mitigation includes patient education on sick-day rules, temporary discontinuation before major surgery, and consideration of individual risk factors such as low-carbohydrate diets, heavy alcohol use, or pancreatogenic diabetes with limited beta cell reserve. Fournier gangrene is extremely rare but has been reported, reinforcing the importance of prompt evaluation of severe perineal symptoms.

Despite robust outcome benefits, access barriers such as out-of-pocket costs and formulary restrictions can limit real-world uptake of SGLT2 inhibitors [29,30].

Research gaps and future directions

Key research priorities include: (1) defining efficacy and safety in populations underrepresented in pivotal trials, including very advanced CKD, dialysis, HFpEF without diabetes, and patients with progressive liver fibrosis; (2) clarifying optimal sequencing and combination with RAAS inhibitors, MRAs, ARNI, and emerging MASH therapeutics; (3) identifying biomarkers that predict response and guide monitoring, such as N-terminal pro b-type natriuretic peptide (NT-proBNP) for HF severity and monitoring, urine albumin-creatinine ratio (uACR) for CKD risk stratification, candidate CKD biomarkers such as fibroblast growth factor-23 (FGF-23), and emerging non-invasive liver fibrosis markers (eg, elastography-based measures and serum fibrosis panels) for hepatic disease; and (4) conducting longer-duration studies that evaluate patient-centered outcomes, including quality of life, functional status, and hard hepatic endpoints. Ongoing long-term surveillance is also needed to refine rare safety risks and to determine the durability of benefit across diverse clinical settings.

Despite their proven clinical benefits, the cost of SGLT2 inhibitors remains a significant barrier to widespread adoption. While these medications can reduce healthcare expenditures by lowering rates of heart failure hospitalization and delaying CKD progression, their high retail prices, often over $500 per month, pose a financial challenge for many patients [29]. Studies indicate that out-of-pocket expenses average around $140 per month, which may contribute to non-adherence or delayed initiation of therapy [29]. Insurance coverage variability and formulary restrictions further limit accessibility, with many patients unable to afford these therapies even when prescribed [30]. In the US, barriers differ by payer type, including commercial insurance, Medicare Part D, and Medicaid, with formulary restrictions and cost-sharing requirements contributing to variable patient out-of-pocket costs and delayed initiation [30]. Research suggests that these affordability concerns influence physician prescribing behavior, potentially hindering the broader utilization of SGLT2 inhibitors despite strong guideline recommendations. Additionally, a nationwide cohort study found that physician adoption of these medications remains inconsistent, highlighting gaps in clinical practice despite endorsements from major health agencies [31]. In resource-constrained settings, limited access emphasizes the need for policy-level strategies and cost-sharing measures to ensure broader availability.

Looking ahead, several areas warrant further exploration to maximize the benefits of SGLT2 inhibitors. Larger, biopsy-confirmed trials are needed to establish robust evidence for their use in NAFLD/NASH. Combination therapies, such as pairing SGLT2 inhibitors with glucagon-like peptide-1 (GLP-1) receptor agonists or MRAs, may offer enhanced efficacy in treatment-resistant HF and advanced CKD. Precision medicine approaches could help identify biomarkers that predict which patients will derive the greatest benefit, particularly in non-diabetic CKD or HFpEF subtypes. Furthermore, additional research is needed to clarify the safety and efficacy of SGLT2 inhibitors in patients with eGFR <20 mL/min/1.73 m² or those requiring dialysis. Addressing these research gaps, alongside implementing cost-reduction strategies, could help improve access and optimize outcomes for patients who stand to benefit most from SGLT2 inhibitor therapy.

Limitations of this review

This manuscript is a narrative review and therefore has limitations compared with a formal systematic review or meta-analysis. Although we used a structured search strategy and prioritized outcomes trials and guidelines, study selection and synthesis were qualitative and subject to publication bias and heterogeneity across study designs, and no formal risk-of-bias tool or GRADE framework was applied. Trial populations differ in baseline risk, eGFR thresholds, background therapy, follow-up duration, and endpoint definitions, which can complicate cross-trial comparisons and may contribute to differences in apparent effect size across studies and outcomes (e.g., hospitalization-driven composites versus mortality endpoints). We did not quantitatively pool estimates; therefore, conclusions rely on concordance across major trials rather than a summary effect. For hepatic disease, much of the evidence remains based on surrogate endpoints with variable definitions of MASLD/MASH and limited long-term outcomes, so conclusions in this domain should be interpreted cautiously.

## Conclusions

SGLT2 inhibitors have emerged as a transformative therapy extending beyond glycemic control to provide significant benefits in HF and CKD. Their mechanisms, ranging from natriuresis to metabolic and anti-inflammatory effects, underscore their broad protective potential across multiple organ systems. Clinical evidence has firmly established their role in reducing hospitalizations, slowing disease progression, and improving overall outcomes in both diabetic and non-diabetic populations.

Despite these advancements, challenges remain in ensuring widespread access due to cost barriers and variability in adoption. Future research should focus on refining patient selection, exploring combination therapies, and further evaluating their role in liver disease. As their use continues to expand, SGLT2 inhibitors represent a shift toward holistic, organ-protective strategies that redefine modern treatment paradigms.
